# Assessing the role of primary healthy microglia and gap junction blocker in hindering Alzheimer’s disease neuroinflammatory type: Early approaches for therapeutic intervention

**DOI:** 10.3389/fnins.2022.1041461

**Published:** 2023-01-10

**Authors:** Mai M. Anwar, Esra Özkan, Narges Shomalizadeh, Selin Sapancı, Ceyda Özler, Judy Kesibi, Yasemin Gürsoy-Özdemir

**Affiliations:** ^1^Department of Biochemistry, National Organization for Drug Control and Research/Egyptian Drug Authority, Cairo, Egypt; ^2^Koç University Research Center for Translational Medicine, KUTTAM, Koç University, Istanbul, Turkey; ^3^Department of Neurology, School of Medicine, Koç University, Istanbul, Turkey

**Keywords:** Alzheimer’s disease, Aβ, CBX, ICV, inflammation, microgliosis, healthy microglia, tau protein

## Abstract

Alzheimer’s disease (AD) is a predominantly heterogeneous disease with a highly complex pathobiology. The presence of amyloid-beta (Aβ) depositions and the accumulation of hyperphosphorylated tau protein remain the characteristic hallmarks of AD. These hallmarks can be detected throughout the brain and other regions, including cerebrospinal fluid (CSF) and the spinal cord. Microglia cells, the brain-resident macrophage type of the brain, are implicated in maintaining healthy brain homeostasis. The localized administration of primary healthy microglia (PHM) is suggested to play a role in mitigating AD hallmark depositions and associated cognitive dysfunction. Carbenoxolone (CBX) is the most common gap junction blocker. It cannot effectively cross the blood–brain barrier (BBB) under systemic administration. Therefore, localized administration of CBX may be a recommended intervention against AD by acting as an antioxidant and anti-inflammatory agent. This study aims to determine whether the localized intracerebroventricular (ICV) administration of PHM and CBX may act as an effective therapeutic intervention for AD neuroinflammatory type. In addition, this study also aims to reveal whether detecting AD hallmarks in the spinal cord and CSF can be considered functional and effective during AD early diagnosis. Male albino rats were divided into four groups: control (group 1), lipopolysaccharide (LPS)-induced AD neuroinflammatory type (group 2), ICV injection of LPS + isolated PHM (group 3), and ICV injection of LPS + CBX (group 4). Morris water maze (MWM) was conducted to evaluate spatial working memory. The brain and spinal cord were isolated from each rat with the collection of CSF. Our findings demonstrate that the localized administration of PHM and CBX can act as promising therapeutic approaches against AD. Additionally, Aβ and tau toxic aggregates were detected in the spinal cord and the CSF of the induced AD model concomitant with the brain tissues. Overall, it is suggested that the ICV administration of PHM and CBX can restore normal brain functions and alleviate AD hallmark depositions. Detecting these depositions in the spinal cord and CSF may be considered in AD early diagnosis. As such, conducting clinical research is recommended to reveal the benefits of related therapeutic approaches compared with preclinical findings.

## Introduction

Inflammation is an innate reaction of the immune system that is directly correlated with infection, alcoholism, depression, anxiety, and autoimmune diseases (Chen et al., 2018; Agarwal et al., 2022). Although inflammation is intended to protect the body against excessive inflammatory reactions, it may cause severe damage to organs and tissues (Fernandes-Alnemri et al., 2009; Lyman et al., 2013). One of the organs that has been reported to be severely affected by inflammation is the brain, even with the presence of a very restrictive blood–brain barrier (BBB). Neuroinflammation is a devastating factor that exhibits common inflammatory features in a wide range of central nervous system disorders (CNS). Such disorders may include multiple sclerosis (MS), amyotrophic lateral sclerosis (ALS), Alzheimer’s disease (AD), and other neurodegenerative diseases (NDS) (Glass et al., 2010; Kempuraj et al., 2016). These triggered inflammatory cascades result in direct immune activation of the brain, which causes collateral damage to brain tissue and irreversible neurodegeneration (Jacobs and Tavitian, 2012).

Alzheimer’s disease is a debilitating and ultimately fatal progressive neuropsychiatric disorder characterized by severe cognitive loss. It is the most prominent and common cause of dementia among the middle aged and elderly (Fitzpatrick et al., 2005; Cavanaugh et al., 2014). The main characteristic AD hallmarks are senile plaques (SPs) and neurofibrillary tangles (NFTs) that cause direct neural degeneration and severe synaptic loss (Nichols et al., 2019). SPs are extracellular deposits of amyloid-beta (Aβ) aggregates. NFTs are intracellular fibrillar aggregates of the tau microtubule-associated protein primarily exhibited by hyperphosphorylation. The accumulation of SPs and NFTs in different brain regions such as the frontal cortex, hippocampus, and basal forebrain results in impaired learning and memory functions (Kinney et al., 2018). Both Aβ and tau protein deposits can extend to the spinal cord and biological fluids, including CSF, which can be used in the early diagnosis of AD (Giacco et al., 2019). Thus, brain regions damaged by AD exhibit serious pathological abnormalities, including synaptic loss, reactive gliosis, and neuronal damage (Wang et al., 2016).

Microglia phenotypes are the main regulators of the innate immune responses in the CNS. Microglia exert various morphological changes in response to pathological factors in the brain. Once activated, microglia cells release various factors such as chemokines, cytokines, and/or anti-inflammatory factors (Thurgur and Pinteaux, 2019; Voet et al., 2019). To perform protective functions, microglia cells adopt different states and phenotypes according to the surrounding environment. This can lead to either neuroinflammation or brain tissue recovery (Li and Barres, 2018). Thus, microglia play the main role in maintaining the neurobiological and neurophysiological homeostatic system of the brain, whether in a diseased or healthy state (Solé-Domènech et al., 2016; Leng and Edison, 2021). Microglia cells exhibit three phenotypes: resting-balanced (M0), pro-inflammatory (M1), and anti-inflammatory (M2) type. As for the resting-balanced M0 phenotype, cells are characterized by a highly branched ramified appearance, giving them a huge ability to adapt according to the surrounding brain parenchyma status. Mainly, they are stimulated *via* inflammation, pathogens, and damage-associated molecular patterns (DAMPs) detected through their extensive recognition systems, including toll-like receptors (TLRs) and pattern recognition receptors (PRRs) (Solé-Domènech et al., 2016). The pro-inflammatory M1 cells are induced by brain injuries, inflammation, and AD hallmark aggregations, leading to microgliosis. Microgliosis is a unique pathological inflammatory action that may result in the activation of the microglia M1 phenotype, leading to neurodegenerative diseases (NDS) (Kempermann and Neumann, 2003; Jayaram and Krishnamurthy, 2021). Mainly, the presence of infection, cellular debris, toxic aggregates, and tumor necrosis factor α (TNF-α) in the surrounding brain environment directly induces the activation of the M1 phenotype and the generation of neuroinflammation (Solé-Domènech et al., 2016). Meanwhile, the anti-inflammatory M2 cells are characterized by extensive homeostatic properties and play a vital role in the recovery processes of damaged brain tissue. It was observed that M2 cells are directly activated by triggered macrophages and the release of anti-inflammatory cytokines, including IL-10. In particular, the M2 phenotype acts by inducing NF-κB, which, in turn, triggers the release of anti-inflammatory cytokines, such as IL-4 and IL-10. As such, the M2 phenotype passively repairs and restores damaged brain tissues by engulfing misfolded or aggregated proteins and dead cells (Solé-Domènech et al., 2016; Guo et al., 2022). In AD, the microglia M1 phenotype was reported to be localized around Aβ deposits. Consequently, neuroinflammation is principally implicated in the pathology of AD (Frank-Cannon et al., 2009; Guo et al., 2022). Long-term activation of the inflammatory M1 phenotype was also observed to result in the massive release of pro-inflammatory factors. This suggests that the aggressive activation of innate immune responses contributes significantly to plaques-induced toxicity *via* TLR4 and the receptor for advanced glycation end products (RAGE) (Buchanan et al., 2010). Primary healthy microglia (PHM) cultures comprise a promising *in vitro* tool for investigating the mechanisms by which these cells combat NDS. *In vitro*, microglia M1 phenotype can be induced by interferon-γ (IFNγ), whereas IL-10 is commonly used to induce microglia M2 phenotype. However, PHM cultures represent microglia cells in a resting-balanced phase (M0) in case of being isolated from healthy rats or mice (Lian et al., 2016; Lin et al., 2017). Therefore, extreme caution may be warranted during preparing the isolation protocol for these highly reactive cells (Lian et al., 2016; Lin et al., 2017). The localized therapeutic intervention of PHM in the non-genetically manipulated induced AD neuroinflammatory model is suggested to lead to extensive healing and neuroregenerative actions. Hence, in the present study, we examined the effect of intracerebroventricular (ICV) injection of the isolated primary healthy microglia cells in a neuroinflammation-induced model of AD to examine their restorative and healing potentials.

The actions of the resting glial cells are suggested to be related to the suppression of neuroinflammation. Thus, resting glial cells may act as a potential therapy for neuronal damage induced by AD hallmark depositions (Wang et al., 2016). Meanwhile, it has been reported that gap junction blockers have therapeutic effects in various experimental models of the NDS, including AD and Parkinson’s disease (PD) (Takeuchi and Suzumura, 2014). Carbenoxolone (CBX) is a type of gap junction blocker and a derivative of glycyrrhetinic acid primarily extracted from the licorice root. CBX is most commonly used for treating serious gastric ulcers (Hellmich et al., 2013). Other pharmacological actions for CBX have been reported, including anti-inflammatory, anticonvulsant, and antioxidant activity. Moreover, the nootropic, neurotrophic, neuroprotective, and anti-inflammatory properties associated with CBX may be related to the inhibition of 11β-hydroxysteroid dehydrogenase (11β-HSD). CBX can also act by stimulating the release of endogenous glucocorticoids and inhibiting the release of 11β-HSD. In addition, CBX can induce the release of heat shock proteins (HSPs) and interfere with AD hallmark depositions (Takeuchi and Suzumura, 2014; Sharma et al., 2017; Zhang et al., 2020). Thus, previous experimental studies have shown that CBX is effective against neuronal death, traumatic brain injury, and cerebral ischemia. It is also suggested that the ICV administration of CBX may act against neuroinflammation due to its anti-inflammatory and antioxidant actions (Leshchenko et al., 2006; Takeuchi and Suzumura, 2014; Sharma et al., 2017). The ICV administration of CBX appears to have greater therapeutic potential than systemic injections. It was previously reported that CBX cannot penetrate the BBB with systemic administration (Leshchenko et al., 2006). Therefore, in light of the outlined nootropic, neurotrophic, and neuroprotective properties, this study explores various aspects of the anti-inflammatory and antioxidant properties of CBX against neuroinflammation and related dysfunctions.

To examine the therapeutic efficiency of PHM and CBX, in addition to finding out whether AD hallmarks can be detected early in the spinal cord and CSF, LPS was used to induce a neuroinflammatory AD-like model. The mechanism involved in the induction of neuroinflammation depends mainly on the activation of pro-inflammatory cytokines related to the LPS-CD14 complex associated with the activation of TLR-4. This in turn activates the microglia phenotype M1, NF-κB, and TNF-α, leading to the release of inflammatory cytokines, resulting in neuroinflammation and cognitive dysfunction (Lee et al., 2008; Ory et al., 2015). A number of reported clinical and epidemiological data support the prevalence of NDS in males over females, indicating the main protective contribution of female sex hormones against NDS. Previously reported preclinical findings demonstrated the neuroprotective role of female sex hormones against AD, whereas androgens have not reported any detected effects (Bourque and Di Paolo, 2022). Consequently, in the present study, male albino rats were used to rule out hormonal effects.

## Materials and methods

### Chemicals

The present study was performed with analytical grade chemicals and kits obtained from the following certified distributors: Dulbecco’s modified Eagle’s medium (DMEM) (Lonza), Bromo-phenol blue (Sigma-Aldrich), trypsin blue (Sigma-Aldrich), EDTA (Sigma-Aldrich), EGTA (Sigma-Aldrich), sodium dodecyl sulfate (SDS) (Sigma-Aldrich), Tris base (Sigma-Aldrich), Triton X (Sigma-Aldrich), Tween (Sigma-Aldrich), methanol (Sigma-Aldrich), sucrose (Sigma-Aldrich), glycine (Melford), and L-glutamine-penicillin-streptomycin solution (Sigma-Aldrich). Lipopolysaccharide (LPS) extracted from *Escherichia coli* 0111:B4 was also purchased from Sigma (Sigma-Aldrich, St. Louis, MO, USA).

### Buffers

Buffers used in this study included phosphate-buffered saline (PBS) (KH_2_PO_4_, NaCl, and Na_2_HPO_4_) (Lonza), Hank’s balanced salt solution (HBSS) without calcium, and magnesium/phenol red (Lonza).

### Isolation and preparation of primary healthy microglia (PHM)

**FIGURE 1:**
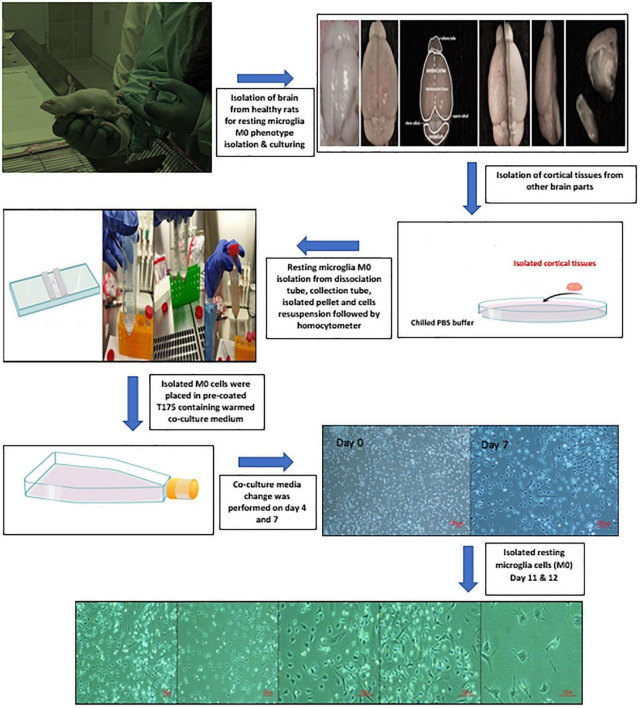
Illustration of primary healthy microglia (PHM) isolation steps and light microscopy images of cultured PHM after 11 and 12 days.

Six male healthy albino rats (15–18 days) were used for the isolation and preparation of PHM cells. Decapitation of the rats at the head/neck junction was performed under anesthesia. Isolated heads were placed in a Petri dish containing chilled PBS. All the brains were isolated from the cranial cavity and placed back in another Petri dish containing chilled PBS. The hemispheres were separated along the median longitudinal fissure, followed by discarding the brainstem, cerebellum, and olfactory bulbs. Meanwhile, the forebrains were kept in the third-chilled PBS for further isolation. Cortical tissues were mainly planned to be used for the isolation and preparation of PHM cells. Thereby, the cortical tissues were placed in a dissociation tube containing HBSS, DNase I Stock Solution, and 10 × trypsin after removing the meninges, midbrain, and hippocampus, respectively, from each forebrain. Using a 10-ml pipette, isolated cortical tissues were pipetted up and down to be crushed into small pieces and incubated in a 37°C water bath for 15 min. Isolated suspension cells were transferred to collection tubes containing fetal bovine serum (FBS), then resuspended in 25 ml of PBS and centrifuged at 300 × *g* for 5 min. Next, 5 ml warmed co-culture media containing 1 × L-glutamine-penicillin-streptomycin solution, 10% FBS in MEM, and filtered 3 mg/ml DNase I in PBS was used to resuspend isolated cells. Cells were counted using a hemocytometer. Then, 1.2 million cells were placed in pre-coated T175 culture flasks containing 45 ml of the warmed co-culture media. Flasks were kept incubated in a 5% CO_2_ humidified incubator and the temperature was maintained at 37°C. A complete change of co-culture media occurred on days 4 and 7. Meanwhile, PHM cells were isolated on day 11 or 12 after being kept on a shaker for 2 h at 180 rpm inside a 5% CO_2_ humidified incubator (37°C) to detach cells (Ni and Aschner, 2010) with certain modifications. The co-culture media was collected from the flasks and passed through a 40-mm cell strainer, then centrifuged at 300 × *g* for 5 min to obtain the desired microglia cells yield and type. Subsequently, isolated PHM cells were observed under a light microscope (Figure 1).

### Experimental design

**FIGURE 2:**
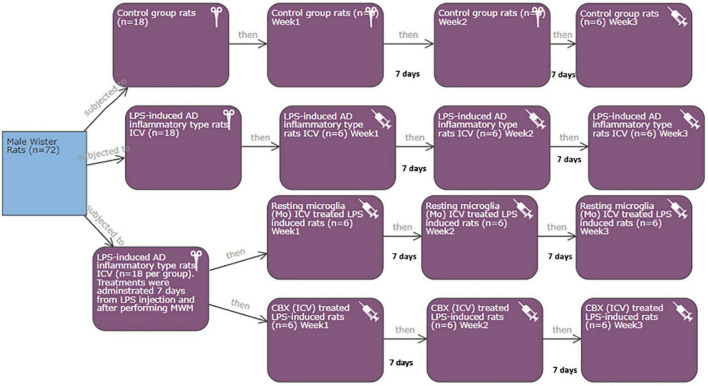
Illustration of the conducted experimental design.

This study was applied according to the required protocols and guidelines of the Koç University Institutional Animal Care and Use Committee (IACUC) in accordance with the directive 2010/63/EU of the European Parliament and Council on the protection of animals used for scientific purposes. Additionally, all experiments were conducted in compliance with the animal research reporting *in vivo* experiments (ARRIVE) guidelines. Rats were housed in plastic cages and kept maintained at a controlled temperature (22°C/12-h light/dark cycle) with free access to food and water. Power calculations: the total number of rats required for the current study was seventy-two male Wistar albino rats (250-/3 months old) divided equally into four groups (eighteen each). Each group was subdivided into three subgroups (6 rats per each) (Figure 2). The minimum and maximum number of rats per group and subgroup were calculated (Charan and Kantharia, 2013; Arifin and Zahiruddin, 2017). Mortality rate (as a result of induction), error types, standard deviation, and the number of tested parameters were all considered during sample size calculation (Charan and Kantharia, 2013; Arifin and Zahiruddin, 2017).

#### Control (group 1)

Eighteen rats were divided equally into three subgroups representing 3-week time interval: control group 1-W1, control group 1-W2, and control group 1-W3.

The rest of the fifty-four male albino rats were ICV injected with LPS. Gram-negative bacterial LPS has previously been proven to induce the non-genetically manipulated neuroinflammatory AD type in rats. Initially, rats were maintained under anesthesia using 2.5% isoflurane mixed with O_2_ at a flow rate of 1 L/min and then injected with ketamine (100 mg/kg, i.p.). Anesthetized rats were monitored for respiration rate, heart rate, and blood pressure. After being completely anesthetized, rats were placed on a stereotaxic frame (Stoelting, Wood Dale, IL, USA), where each rat received a single ICV injection of LPS (2 μl) at a flow rate of 0.5 μl/min. The following positions were used in relation to the bregma: anterior-posterior (AP) = −3.6, lateral-medial (LM) = ± 2.0, and dorsal-ventral (DV) = −3.4 (Espinosa-Oliva et al., 2013b; Jangra et al., 2021) with certain modifications. Induced rats were monitored during the recovery period and kept under a heat lamp regularly until they were completely conscious and active (Espinosa-Oliva et al., 2013b). The total period of AD induction following the ICV LPS injection was 7–10 days. Within hours of LPS injection, motion sickness and behavioral changes were observed as a result of the induction of AD-like symptoms. The fifty-four LPS-induced AD neuroinflammatory rats were divided as follows:

#### LPS-induced AD neuroinflammatory type (group 2)

Eighteen neuroinflammatory AD-induced rats were divided equally into three subgroups. The three subgroups represent a 3-week time interval following the ICV injection of LPS: neuroinflammatory AD-induced group 2-W1, neuroinflammatory AD-induced group 2-W2, and neuroinflammatory AD-induced group 2-W3.

#### LPS-induced AD neuroinflammatory rats received a single intracerebroventricular injection of PHM cells (group 3)

Other eighteen male neuroinflammatory AD-induced rats received a single ICV injection of the isolated PHM (2 μl) (∼10,000 cells) (Kitamura et al., 2004; Espinosa-Oliva et al., 2013a; Jangra et al., 2021) with certain modifications. Group 3 was conducted to determine the efficacy of a single ICV injection of PHM cells as a successful therapeutic intervention among AD-induced rats. Thereby, group 3 was subdivided into three subgroups. The three subgroups represent a 3-week time interval following the single ICV injection of PHM cells: microglia-treated LPS-induced group 3-W1, microglia-treated LPS-induced group 3-W2, and microglia-treated LPS-induced group 3-W3.

#### LPS-induced AD neuroinflammatory rats received a single ICV injection of CBX (group 4)

The remaining eighteen male neuroinflammatory AD-induced rats received a single ICV injection of 25 μg/kg (CBX). The following positions were used in relation to the bregma: AP = −3.6, LM = ± 2.0, and DV = −3.4 (Khorasani et al., 2009; Espinosa-Oliva et al., 2013a; Jangra et al., 2021) with certain modifications. Group 4 was conducted to determine the efficacy of a single ICV injection of CBX as a therapeutic intervention among AD-induced rats. Thereby group 4 was successfully subdivided into three subgroups. The three subgroups represent a 3-week time interval following the single ICV injection of CBX: CBX-treated LPS-induced group 4-W1, CBX-treated LPS-induced group 4-W2, and CBX-treated LPS-induced group 4-W3.

### Morris water maze (MWM) behavioral assessment

At the end of the experiment and prior to conducting further biological assessment procedures, all rats in all subgroups were assigned to MWM. The purpose of MWM was to evaluate the extent of behavioral changes in the LPS-induced AD neuroinflammatory model. Rats were placed in a circular pool filled with opaque water at a controlled temperature of 25°C. A circular platform was submerged 1 inch under the water in the middle of the target quadrant. Rats were trained for three consecutive days, each lasting 60 s per rat. On day 7, a final test was conducted where the time taken by each rat to reach the platform was directly recorded and calculated (Anwar et al., 2021).

### Perfusion and sample preparation

The CSF was obtained from each rat directly following the MWM assessment and prior to infusion by the direct puncture of the cisterna magna using a 24-gauge insulin syringe (0.127 needle wall thickness) under anesthesia. Following perfusion with 10 IU/L saline and 4% ice-cold heparinized paraformaldehyde (PFA), the brain and the spinal cord were isolated from each rat (Richner et al., 2017). The collected CSF and the isolated brain and spinal cord of each rat were used for further biological evaluation in addition to histopathological studies and immunofluorescence staining.

### ELISA

Aβ1-42 and pMAPT/pTAU (phosphorylated microtubule-associated protein tau) were detected among brain and spinal cord tissue homogenates in addition to CSF. Thus, brain–spinal cord tissue homogenates and CSF were prepared and assessed according to the manufacturer’s instructions. Aβ1-42 and pMAPT/pTAU were both purchased from Elabscience (catalog no: E-EL-R1402 and E-EL-R1090, respectively).

### Histopathological examination, Congo red, and immunofluorescence staining

The isolated brain and spinal cord of each rat were directly kept in 4% PFA (4°C) overnight to prepare the isolated tissues for hematoxylin and eosin (H&E), Congo red (CR), and immunofluorescence staining. The brain parts and spinal cord were dehydrated by a respective gradient of 10, 20, and 30% of sucrose in 0.1 M phosphate buffer (4°C). H&E and CR staining were normally applied on the paraffin-embedded brain and spinal cord sections. For CR staining, sections were rehydrated with a series of high-grade ethanol and water. Slides were then incubated in 50 ml of 50% ethanol and 0.01% sodium hydroxide (NaOH) for 5 min. Subsequently, slides were incubated in 0.5% CR solution for 20–30 min. Following CR solution incubation, sections were directly incubated in 50 and 70% ethanol one time for 1 min each. This was followed by rinsing and incubating the slides with 95 and 100% ethanol 2 times for 2 min each. Finally, slides were rinsed with xylene 2 times for 3 min before cover slipping in DPX mounting medium (Sigma-Aldrich, St. Louis, MO, USA). For immunofluorescence, tissues were embedded in Cryomatrix embedding resin and were directly sliced at a thickness of 30 μm. Embedded slides were immersed directly in distilled water and soaked in methanol for 5 min, then washed with Dulbecco’s phosphate-buffered saline (DPBS). These slides were then blocked in a commercial Super Block solution for 1 h at room temperature. Additionally, blocked slides were incubated separately with Anti-glial fibrillary acidic protein (GFAP) (SAB5700611, Rabbit) and Anti-NeuN (ABN78C3, Rabbit) primary antibodies (Sigma Aldrich, 1:100 dilution) at 37°C for 90 min. After washing the slides with DPBS, they were directly incubated with Goat anti-rabbit-cy3 secondary antibodies (diluted in 1:200 Super Block) for 90 min at 37°C in the dark. Finally, each slide was mounted with a 4,6-diamidino-2-phenylindole mounting medium (DAPI, Abcam; Ab104139). Immunofluorescence labeling of the prepared slides was examined using a Leica DMI8 SP8 confocal live scanning microscope.

### Image analysis

Obtained fluorescence microscopy images were directly exported as tiff files using the LASX program (Leica, Wetzlar, Germany). To quantify the number and percentage area of the cells, the ImageJ program was used.

### Statistical analysis

The data were expressed as mean ± SD and analyzed using the GraphPad Prism version 5.0 and SPSS version 25 software. The differences among groups and within a different time interval (3 weeks) were analyzed using single-repeated measurement ANOVA followed by the Bonferroni *post-hoc* test adjustment. The results were considered significant at *p* < 0.05. The GraphPad Prism version 5.0 software was used to draw graphs.

## Results

### MWM behavioral and cognitive impairment assessment

**FIGURE 3:**
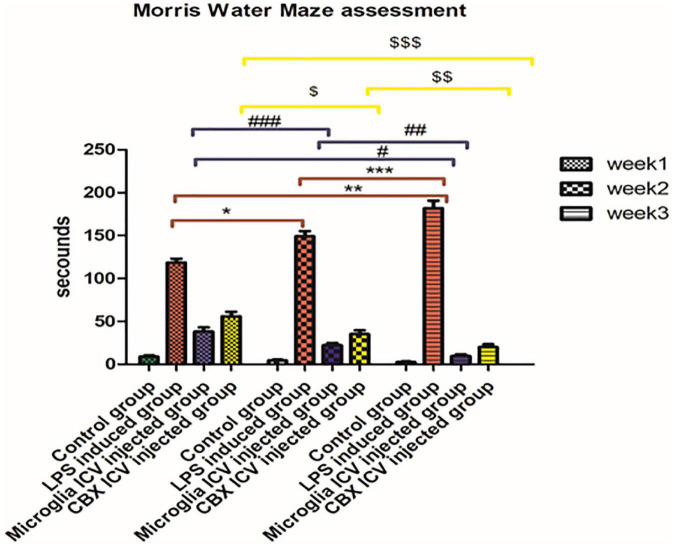
Latency time in seconds taken by each rat to reach the platform in the Morris Water Maze (MWM). Represented data were expressed as mean ± SD (*n* = 6 per group) (ANOVA, repeated measurement, Bonferroni *post-hoc* test, *p* < 0.05). The same asterisks represent an insignificant difference *p* > 0.05.

Morris water maze (MWM) was assessed to ensure the induction of cognitive dysfunction. Our results revealed an observed memory loss in LPS-induced rats. This was represented by the time taken by each rat to reach the hidden platform (latency time) compared with the control groups (Figure 3). A cascading increase in calculated latency time was observed in LPS-induced rats during weeks 1, 2, and 3. The longest latency period was observed in LPS-induced rats, week 3, compared with the control groups (*p* < 0.05) (Figure 3). Following the single ICV injection of PHM and CBX, a relevant decrease in the latency time was detected in all the treated groups over the 3-week time interval compared with the control groups (Figure 3). Meanwhile, the observed results indicate that the PHM-treated group (week 3) had the least latency time in the MWM, followed by the CBX-treated group (week 3). Thus, these findings showed that the ICV administration of PHM gives a better action in the long term compared to the control group (week 3) (*p* < 0.05). These PHM actions may be related to the microgliosis hindering phenomena (Figure 3). On the other hand, the ICV administration of CBX designated its efficacy when administered locally in the brain. This indicates its promising efficacy in lessening neuroinflammation compared with the control group (week 3) (*p* < 0.05) (Figure 3).

### Relieved Aβ hallmark depositions following the ICV administration of PHM and CBX

**FIGURE 4:**
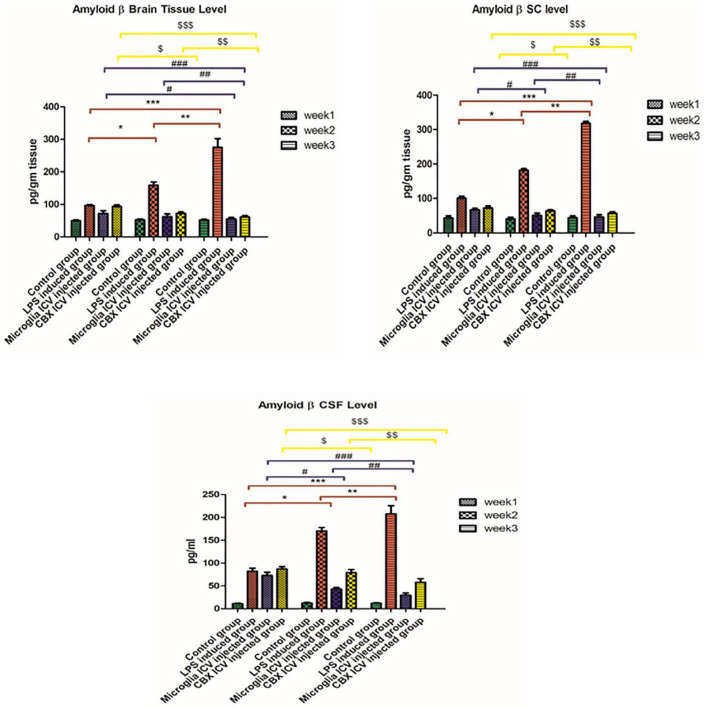
Illustration of Aβ expression levels in brain tissue, spinal cord, and cerebrospinal fluid (CSF) in the lipopolysaccharide (LPS)-induced rats and following the single intracerebroventricular (ICV) administration of primary healthy microglia (PHM) and carbenoxolone (CBX) over the 3-week time interval. Represented data were expressed as mean ± SD (*n* = 6 per group) (ANOVA, repeated measurement, Bonferroni *post-hoc* test, *p* < 0.05). The same asterisks represent an insignificant difference *p* > 0.05.

Our results indicate that Aβ depositions were detected in the brain, spinal cord, and CSF of the LPS-induced rats, but in a different ratio. The highest Aβ level was found in the brain and spinal cord of the LPS-induced group (week 3). In addition, a less Aβ expression level was detected in the CSF of the LPS-induced group (week 3) relative to the control group (*p* < 0.05) (Figure 4). Meanwhile, the single ICV administration of PHM relieved and engulfed Aβ depositions in all isolated tissues and CSF. The most significant therapeutic effect was detected in the brain and spinal cord of the PHM-treated group (week 3) compared with the control group (*p* < 0.05) (Figure 4). Additionally, the ICV administration of CBX demonstrated an Aβ engulfing potency compared with the control group. This observed decrease in Aβ level is not equivalent to that observed in the microglia-treated groups, especially in the CSF. Thus, results show that the administration of PHM effectively retained the acceptable level of Aβ in the brain and spinal cord compared with the control group (*p* < 0.05) (Figure 4), suggesting that it could be a therapeutic intervention.

### Restoring potentials of the PHM and CBX ICV injection on tau protein homeostasis level

**FIGURE 5:**
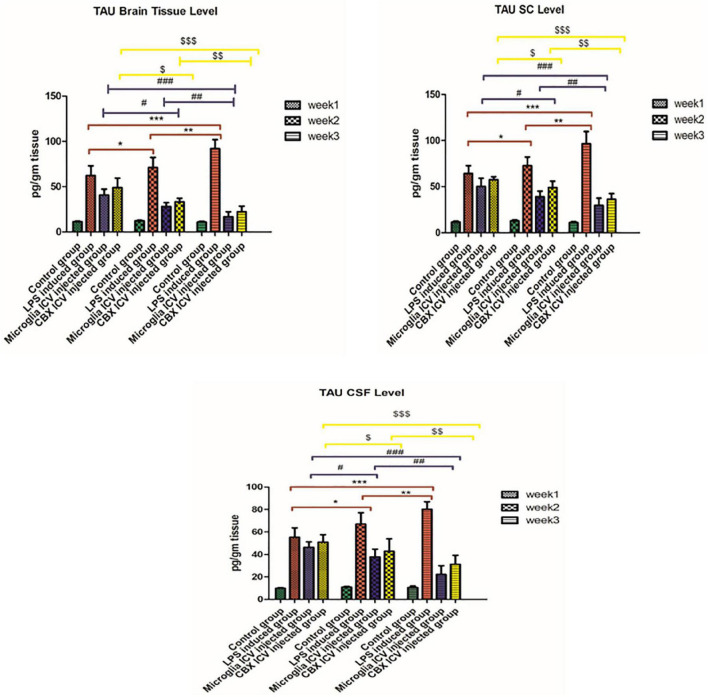
Illustration of tau protein expression levels in brain tissue, spinal cord, and cerebrospinal fluid (CSF) in the lipopolysaccharide (LPS)-induced rats and following the single intracerebroventricular (ICV) administration of primary healthy microglia (PHM) and carbenoxolone (CBX) over the 3-week time interval. Represented data were expressed as mean ± SD (*n* = 6 per group) (ANOVA, repeated measurement, Bonferroni *post-hoc* test, *p* < 0.05). The same asterisks represent an insignificant difference *p* > 0.05.

Our results demonstrate that the ICV injection of LPS resulted in a massive increase in tau protein levels during weeks 1, 2, and 3 (*p* < 0.05) (Figure 5). The effective potential therapeutic potency of PHM was demonstrated by the decreased tau protein levels in the brain, spinal cord, and CSF following the single ICV administration. However, the best effect was detected in week 3 relative to the control group (*p* < 0.05) (Figure 5). These observed results indicate that the efficiency of microglia administration increases over time (*p* < 0.05) (Figure 5). CBX also demonstrated significant therapeutic potential by decreasing the elevated tau protein levels in the brain, spinal cord, and CSF compared with the control group (*p* < 0.05) (Figure 5). Meanwhile, CBX therapeutic potentials against tau protein were found to be more effective in the brain than in the spinal cord and CSF, especially in week 3. Thus, the efficiency of CBX against tau protein spinal cord and CSF levels was less effective than in the brain compared with the control group (week 3) (*p* < 0.05) (Figure 5).

### Aβ CR staining depletion potential following the ICV administration of PHM and CBX

**FIGURE 6:**
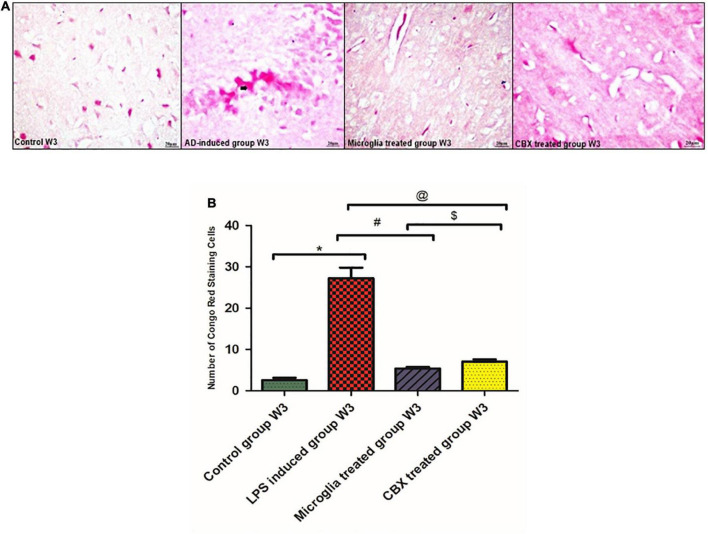
Illustration of Congo red (CR)-stained brain sections of the lipopolysaccharide (LPS)-induced rats and following the intracerebroventricular (ICV) administration of primary healthy microglia (PHM) and carbenoxolone (CBX) in week 3 for the visualization of Aβ. **(A)** Illustrative images of positive Aβ hallmark depositions stained by CR in the LPS-induced AD neuroinflammatory model and following the single ICV administration of PHM and CBX (week 3). **(B)** Number of CR positively stained cells, where the observed stained Aβ cells were counted under the light microscope. Represented data were expressed as mean ± SD (*n* = 6 per group) (ANOVA, repeated measurement, Bonferroni *post-hoc* test, *p* < 0.05). The same asterisks represent an insignificant difference *p* > 0.05.

Congo red (CR) staining was used to indicate Aβ burden deposits on neurons. The presented results show that the single ICV administration of LPS resulted in an increased number of Aβ-stained cells in the brain (week 3) compared with the control group (*p* < 0.05) (Figure 6). Meanwhile, the ICV administration of PHM and CBX resulted in the depletion of Aβ-stained cells. A more significant detected progression was observed in the microglia-treated group (week 3) than in the CBX-treated group (week 3) compared with the control group (*p* < 0.05) (Figure 6).

### Intracerebroventricular administration of PHM and CBX mitigated the histopathological and morphological damages induced by neuroinflammation

**FIGURE 7:**
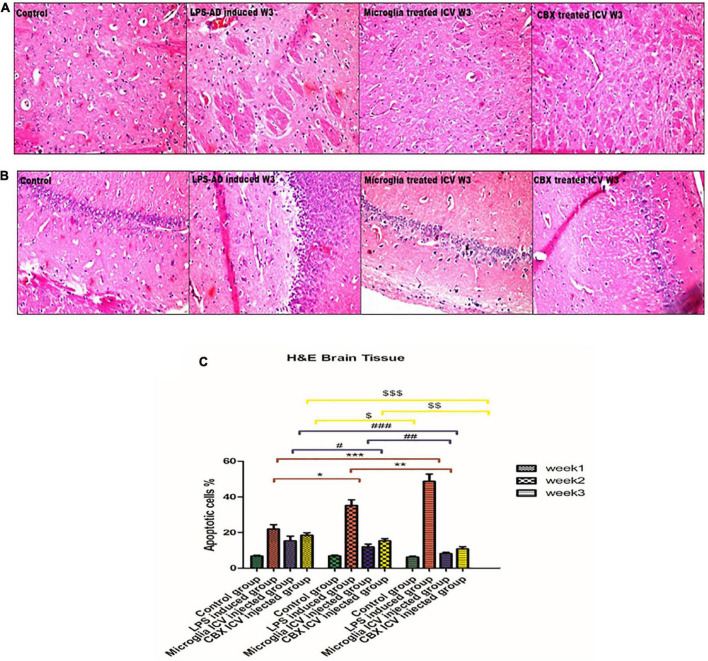
Histopathological examination and assessment of the cerebral cortex and hippocampus stained H&E sections of the lipopolysaccharide (LPS)-induced rats and following the single intracerebroventricular (ICV) administration of primary healthy microglia (PHM) and carbenoxolone (CBX) (week 3). **(A,B)** Detection of the cerebral cortex and hippocampus H&E-stained sections in the control, LPS-induced group, PHM-treated group, and CBX-treated group (week 3). **(C)** Percentage of apoptotic cells histogram over the 3-week time interval. Represented data were expressed as mean ± SD (*n* = 6 per group) (ANOVA, repeated measurement, Bonferroni *post-hoc* test, *p* < 0.05). The same asterisks represent an insignificant difference *p* > 0.05.

**FIGURE 8:**
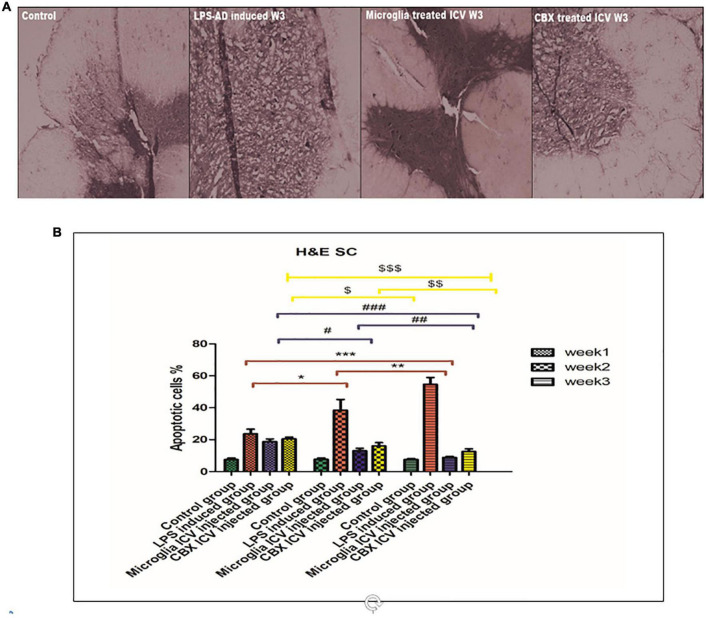
Histopathological examination and assessment of the spinal cord stained H&E sections of the lipopolysaccharide (LPS)-induced rats and following the single intracerebroventricular (ICV) administration of primary healthy microglia (PHM) and carbenoxolone (CBX) (week 3). **(A)** Detection of the spinal cord-stained sections in the control, LPS-induced group, PHM-treated group, and CBX-treated group (week 3). **(B)** Percentage of apoptotic cells histogram over the 3-week time interval. Represented data were expressed as mean ± SD (*n* = 6 per group) (ANOVA, repeated measurement, Bonferroni *post-hoc* test, *p* < 0.05). The same asterisks represent an insignificant difference *p* > 0.05.

Sections of the brain and spinal cord were isolated and stained with H&E to evaluate the efficacy of the ICV administration of PHM and CBX in AD-induced rats (Figures 7, 8). Initially, the effects of LPS induction were detected and analyzed for 3 weeks in comparison with the control groups. Exposure to LPS resulted in Aβ depositions, pyramidal neuronal swelling, a decrease in the number of neurons, and an observed increase in apoptotic cells. Additionally, cell clumping and prominent degenerative hemorrhage patches were mainly detected in week 3 compared with the control group (*p* < 0.05) (Figure 7). Apoptotic cells were identified by having clusters of oval bodies, an intense eosinophilic cytoplasm, a lack of nuclear membrane integrity, pyknosis, and karyorrhexis (Wyllie et al., 1980; Majno and Joris, 1995). The total number of apoptotic cells was calculated as follows: (Number of detected apoptotic neuronal cells/Total number of healthy cells nuclei in brain section) × 100 (Yan et al., 2017).

These findings indicate that LPS injection directly led to cellular damage and cascading degenerative structural changes. On the other hand, treatment of LPS-exposed rats with PHM and CBX repaired the induced morphological damage in brain tissue. Meanwhile, a more significant efficiency was detected following PHM administration compared to the control groups (*p* < 0.05) (Figure 7). Results revealed that PHM and CBX exert their maximum healing effects in week 3 compared to weeks 1, and 2. The histopathological evaluation of the spinal cord sections of the LPS-exposed groups revealed a severe motor neuron loss with a lot of cavities, disarranged nerve fibers, liquefaction, and neuronal atrophy over the 3-week time interval (Figure 8). The results also demonstrated that axonal remyelination, a significant reduction in cavitation area, liquefaction, and apoptotic cell number were all detected following the ICV administration of PHM and CBX. Meanwhile, significantly recovered spinal cord sections were observed following PHM administration, especially in week 3 (*p* < 0.05) (Figure 8). Apoptotic cells were identified by having clusters of oval bodies, an intense eosinophilic cytoplasm, a lack of nuclear membrane integrity, pyknosis, and karyorrhexis (Wyllie et al., 1980; Majno and Joris, 1995). The total number of apoptotic cells was calculated as follows: (Number of detected apoptotic neuronal cells/Total number of healthy cells nuclei in spinal cord section) × 100 (Yan et al., 2017).

### Attenuating effect of PHM and CBX intracerebroventricular administration on exaggerated glial fibrillary acidic protein expression in LPS-exposed rats

**FIGURE 9:**
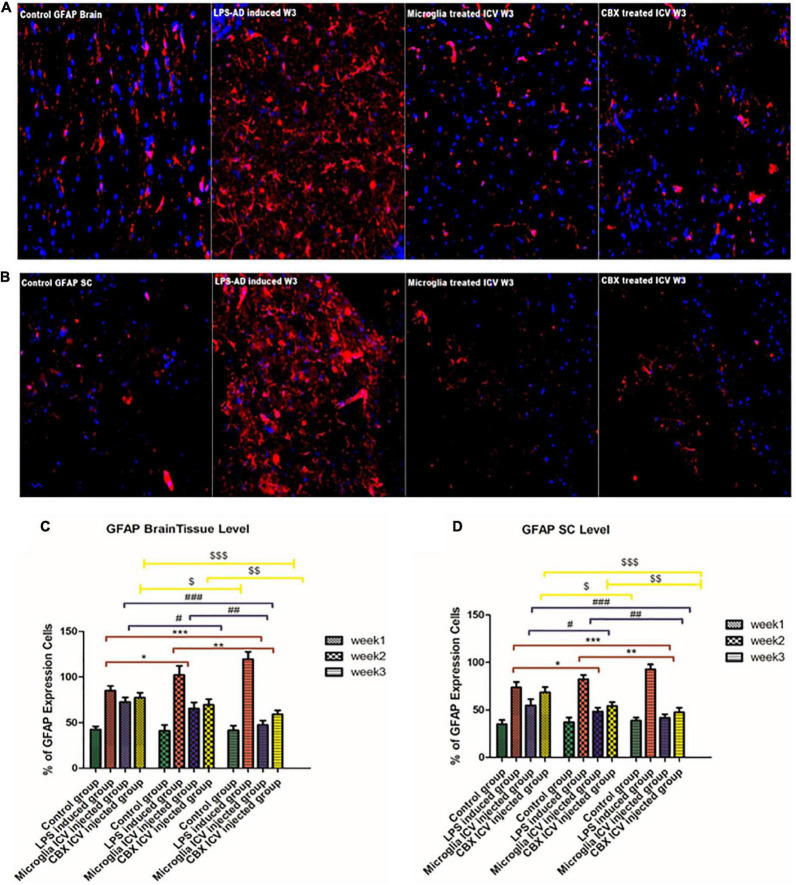
Detection of glial fibrillary acidic protein (GFAP) expression level in the cerebral cortex **(A)** and the spinal cord **(B)** of the lipopolysaccharide (LPS)-induced rats and following the single intracerebroventricular (ICV) administration of primary healthy microglia (PHM) and carbenoxolone (CBX) (week 3). Increased GFAP cell expression was detected in the cerebral cortex and spinal cord sections of the LPS-induced AD neuroinflammatory model. Meanwhile, an observed decrease in the percentage of GFAP expression level was detected in the cerebral cortex and spinal cord sections following the single ICV administration of primary healthy microglia (PHM) and CBX (week 3). **(C)** Illustration of GFAP % expression cells level and quantitative analysis in the cerebral cortex over the 3-week time interval. **(D)** Illustration of GFAP % expression cells level and quantitative analysis in the spinal cord over the 3-week time interval. Represented data were expressed as mean ± SD (*n* = 6 per group) (ANOVA, repeated measurement, Bonferroni *post-hoc* test, *p* < 0.05). The same asterisks represent an insignificant difference *p* > 0.05.

GFAP, an astrocyte marker, was found to be highly expressed in different regions of the brain and spinal cord of the LPS-induced groups over the 3-week time interval compared with the control groups (Figure 9). Expression of GFAP in control groups was found to be stable across the brain and spinal cord regions (Figure 9). It was observed that this excessive GFAP expression observed in the LPS-induced AD groups was significantly reduced following the ICV administration of PHM and CBX over the 3-week time interval. Meanwhile, a more significant decrease in the expression level of GFAP was detected following the PHM administration, especially in week 3 (Figure 9). These results reveal that both PHM and CBX may abate inducing astrocyte activation over the long term. Meanwhile, the best therapeutic effect was detected in week 3 following the single ICV administration of PHM and CBX. The detected restoration level of GFAP expression in the brain and spinal cord of the treated groups was observed to be significantly lower than in the control groups (*p* < 0.05) (Figure 9). On the other hand, a very highly significant improvement was also observed in the PHM- and CBX-treated groups (week 3) with respect to the LPS-induced groups (*p* < 0.05) (Figure 9).

### PHM and CBX improve LPS-induced neuronal impairments and cognitive dysfunction

**FIGURE 10:**
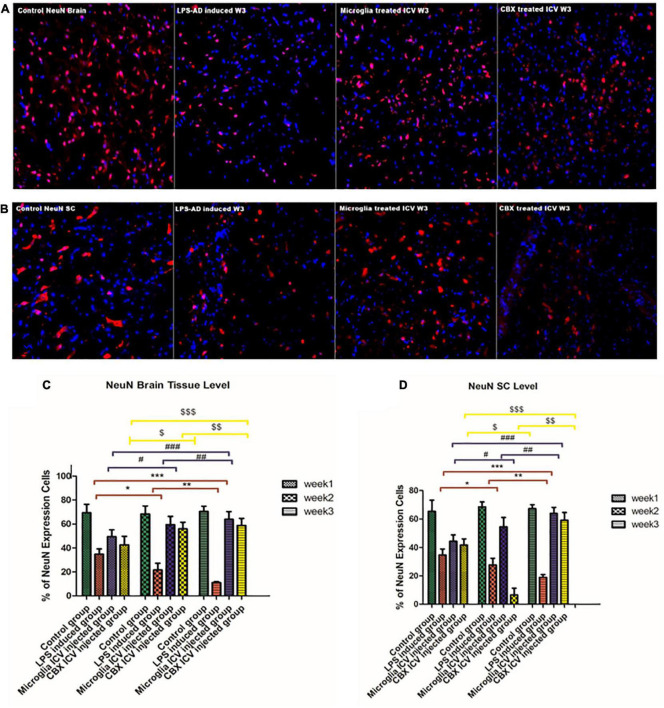
Detection of NeuN expression level in the cerebral cortex **(A)** and the spinal cord **(B)** of the lipopolysaccharide (LPS)-induced rats and following the single intracerebroventricular (ICV) administration of primary healthy microglia (PHM) and carbenoxolone (CBX) (week 3). Decreased NeuN cell expression was detected in the cerebral cortex and spinal cord sections of the LPS-induced AD neuroinflammatory model. Meanwhile, an observed increase in the percentage of the NeuN expression level was detected in the cerebral cortex and spinal cord sections following the single ICV administration of primary healthy microglia (PHM) and CBX (week 3). **(C)** Illustration of NeuN% expression cells level and quantitative analysis in the cerebral cortex over the 3-week time interval. **(D)** Illustration of GFAP % expression cells level and quantitative analysis in the spinal cord over the 3-week time interval. Represented data were expressed as mean ± SD (*n* = 6 per group) (ANOVA, repeated measurement, Bonferroni *post-hoc* test, *p* < 0.05). The same asterisks represent an insignificant difference *p* > 0.05.

The cellular functionality of LPS-induced rats following the ICV administration of PHM and CBX was evaluated by NeuN immunohistochemistry (IHC) staining. Results indicate that LPS induced numerous degenerative changes in different areas of the brain and spinal cord (Figure 10). Thus, the main purpose of IHC NeuN staining was to analyze the degree of neuropathological damage by highlighting the physiological status of neuronal cells in different areas of the brain and spinal cord. NeuN expression was found to be significantly suppressed in the cerebral cortex of LPS-induced rats, with most of the remarkable suppressed expression level detected during week 3 (*p* < 0.05) (Figure 10). Improved and restored NeuN expression was highly notable in PHM- and CBX-treated groups, especially in week 3. Meanwhile, the NeuN expression level was observed to be most significantly relieved following PHM administration compared with the control groups (week 3) (*p* < 0.05) (Figure 10). The detected relieved NeuN expression level in the brain and spinal cord of the different treated groups was observed to be significantly lower than in the control groups (p < 0.05) (Figure 10). On the other hand, a very highly notable improvement was detected in the PHM- and CBX-treated groups (week 3) with respect to the LPS-induced groups (*p* < 0.05) (Figure 10).

## Discussion

Several epidemiological and genetic studies have shown that exaggerated inflammatory cascades can contribute to long-term irreversible AD pathology. It is thus important to build a direct contact between inflammation and promising therapeutics to establish a potentially compelling theoretical link between neuroinflammatory responses and microgliosis. Our results show that the ICV injection of LPS resulted in behavioral changes, memory impairments, and cognitive malfunctions 7 days following injection. After the single injection of LPS, a storm of inflammatory cascades is triggered by TLR-4, CD14, and DAMPS. This is followed by activation of the NF-κB and MAP kinase pathways, leading to the release of inflammatory cytokines and apoptosis. The liberated inflammatory and apoptotic factors cause direct damage to different areas of the brain and spinal cord, including Aβ toxic aggregates (Batista et al., 2019; de Araújo et al., 2022). It was previously reported that the degree of neuropathological damage induced by neuroinflammation is mainly controlled by the type of activated microglial phenotype. This activation involves a polarization shift from M1 pro-inflammatory (classical activation) to M2 anti-inflammatory (alternative activation phenotype) or vice versa in response to the surrounding stimuli and disturbances (Zhang et al., 2019). To outline the activation of both classical and alternative microglia phenotypes, it is important to describe the main function of each phenotype to emphasize their exact role. Microglia are the first defense mechanism, acting as a responder against inflammation, infection, and injury by producing inflammatory cytokines, including TNF-α and IL-6 associated with chemokines (Mills et al., 2000; Zhang et al., 2019). These produced cytokines act as a driving force for the microglia polarization shift toward the M1/TH1 phenotype also known as the classical activation. However, the pro-inflammatory M1 phenotype is suggested to cause brain lesions in response to highly expressed inflammatory cytokines. These expressed cytokines may be involved in hindering inflammation and restoring brain homeostasis (M1–M2 balance). This can be achieved by activating the release of anti-inflammatory cytokines as part of a neuromodulatory response. Thus, the release of IL-10 in the brain mainly results in a direct shift from the M1 phenotype to the M2/TH2 phenotype termed alternative shift (Martinez et al., 2009; Anwar, 2021b). On the other hand, the resting-balanced microglia phenotype (M0) with ramified appearance is the main type of microglia residing in the parenchyma of the healthy brain. Depending on the type of the exposed environment and the identified stimulus, M0 implies different phenotypes, whether M1/TH1 or M2/TH2 phenotype (Guedes et al., 2018; Ji et al., 2019; Leng and Edison, 2021).

Alzheimer’s disease is highly characterized by cognitive dysfunction associated with the accumulation of characteristic hallmarks, including extracellular Aβ plaques and intracellular hyperphosphorylated tau protein (Block et al., 2007; Anwar and Özkan, 2021). These characteristic hallmarks are used in the early diagnosis of AD and act as a driving neuroinflammatory factor leading to neuronal death and cerebral atrophy (Block et al., 2007; Selkoe, 2011; Anwar, 2021a). Our results revealed that following the induction of AD inflammatory type in rats by the single ICV injection of LPS, both types of AD hallmark depositions were recognized in different regions, including the cerebral cortex, hippocampus, spinal cord, and CSF. The amount of expressed Aβ and tau protein was detected comparatively from week 1 to week 3. The most abundant expression was found in the brain tissues, spinal cord, and CSF of the LPS-induced rats (week 3). These observed findings showed that hallmark aggregations are not limited to the brain. As a result, Aβ and tau protein aggregations can be detected in the spinal cord and CS biological fluid and may be used in the early diagnosis of AD. Furthermore, these toxic aggregations had an impact on rat behavior and cognitive functions in MWM. A direct correlation was observed between the increased hallmark depositions and cognitive dysfunction in the brain, spinal cord, and CSF. This was also reflected by the latency time of rats in the MWM. LPS-induced rats in week 3 were observed to consume the longest latency of any participant group compared with controls. It was previously reported that microglia activation during neurodegenerative progression leads to the release of pro-inflammatory cytokines and brain lesions. Thus, several studies have attempted to eliminate and deplete microglia M1 phenotype in neurodegenerative models (Flores-Martinez and Fernandez-Parrilla, 2018). Previous results indicated that the release of inflammatory cytokines in the brain can occur independently of microglia M1 activation (Flores-Martinez and Fernandez-Parrilla, 2018). It is suggested that various important signals and pathways are mainly involved in the microglia polarization from M1 to M2. Therefore, the components of these triggered signals are likely to affect the phenotype of microglia dominating the brain during inflammation (Flores-Martinez and Fernandez-Parrilla, 2018; Guo et al., 2022). It is also suggested that the concept of normalizing microglial pro-inflammatory activities by increasing healthy resting microglia phenotype M0 in the brain may play important role in maintaining brain homeostasis M1–M2 balance. It may even result in a direct shift toward the M2 neuroprotective phenotype (Flores-Martinez and Fernandez-Parrilla, 2018; Anwar and Fathi, 2022; Guo et al., 2022).

Systemic treatments that reverse or prevent AD are ineffective; consequently, various molecular and cellular rehabilitation therapies have been tested in various animal models. One of the main objectives of this study was to determine whether the ICV microglia administration is unambiguously effective in eliminating AD-related damages. The MWM behavior test, brain hallmark deposition, NeuN, and GFAP expression were all evaluated following the single PHM transplantation over the 3-week time interval following 7 days of LPS injection. Statistically significant differences were detected between weeks 1 and 3 in the microglia-treated groups. The best therapeutic effect was observed in the rats at week 3 where they had the shortest latency time in the maze. Consequently, the PHM ICV injection was found to exert a positive impact on restoring normal cognitive functions. A significant decrease in the expression levels of Aβ, tau protein, and GFAP in the brain, spinal cord, and CSF was also observed. Furthermore, an increase in the NeuN expression level was also detected. This suggests that ICV administration of PHM in AD-induced rats, where the M1 phenotype is activated, led to the normalization of pro-inflammatory microglia activity. This was possibly mediated by shifting microglia M1 phenotype to M2 phenotype to maintain cerebral hemostatic balance (Kitamura et al., 2004; Takata et al., 2007, 2022; Narantuya et al., 2010). Such shift from M1 to M2 is suggested to be mediated by signals, stimuli, and released inflammatory substances. Thus, it is suggested that the administrated PHM was stimulated by the exaggerated inflammatory cytokines found in the neuroinflammatory AD-like model. This led to a direct shift toward the M2 anti-inflammatory microglia phenotype. Moreover, this mediated shift resulted in hindering microgliosis and healing of damaged tissue by Aβ phagocytosis/clearance, the release of anti-inflammatory cytokines, and increased brain-derived neurotropic factors (BDNFs). This resulted in the regeneration of damaged brain tissue, structure repair, and neurogenesis, which can be described as balanced microglia-mediated neurogenesis (Kitamura et al., 2004; Takata et al., 2007, 2022; Narantuya et al., 2010).

Since the main negative effects of Aβ aggregations in the brain are increased oxidative stress, behavioral changes, and cognitive decline, all these manifestations may be mainly related to neuroinflammation. Thus, our goal was to find a drug that can hinder or inhibit the neuroinflammatory cascades triggered by a neuroinflammatory type of AD. It was previously reported that CBX can exert a potential anti-inflammatory and neuroprotective effect against AD. The major concern is that CBX cannot penetrate the BBB (Leshchenko et al., 2006; Volnova and Tsytsarev, 2022). Thereby, in this study, a single ICV injection of CBX was administrated to LPS-induced AD inflammatory rats. Our findings show that the localized CBX administration improved behavioral outcomes and mediated anti-inflammatory actions by alleviating Aβ depositions in different brain regions, the spinal cord, and CSF. Additionally, reduced astrocyte activation and increased neurogenesis were observed by reduced GFAP and increased NeuN expression levels. The most significant CBX effect was detected in the CBX-treated group (week 3) with respect to the control groups. On the other hand, our results demonstrate that the ICV injection of CBX significantly increased NeuN expression in the spinal cord, but not effectively as in the brain. Whereas a sudden decrease in the NeuN spinal cord expression level of the CBX-treated group (week 2) was recognized. CBX was found to be effective on the spinal cord during the induction phase (week 1) and the healing/regeneration phase (week 3). It was found not to be very effective during maintenance (week 2). Thus, these results suggest the presence of a critical-time window in which CBX may maintain neurogenesis or prevent neurodegeneration in the spinal cord due to the presence of pro-inflammatory or anti-inflammatory cytokines. This highlights the importance of monitoring the time-dependent drug administration period for managing NDS (Roh et al., 2010). Thus, the healing and regenerative actions of CBX can be related to its efficacy against exaggerated inflammatory cytokines, increased expression of BDNF, reduced Bax apoptotic factor, reduced ACHE level, and hindered oxidative stress release (Khorasani et al., 2009; Hellmich et al., 2013; Thakur and Nehru, 2015; Sharma et al., 2017, 2019). Thus, the observed results underline the effectiveness of the ICV administration of CBX in restoring the survival index of the normal neuronal population and improving cognitive functions (Khorasani et al., 2009; Hellmich et al., 2013; Thakur and Nehru, 2015; Sharma et al., 2017, 2019).

### Study limitations

Despite all our observed results, the limitations of the study can be described as follows: only samples of brain tissue, SC, and CSF were used. Moreover, the AD-induced female model was excluded from the study. As mentioned earlier, only the AD-induced male model was used to avoid the female hormonal interface with our obtained results. Meanwhile, samples from the brain, spinal cord, and CSF were only used to evaluate the effectiveness of the ICV administration of PHM and CBX against LPS-induced AD neuroinflammatory type. Therefore, both male and female AD-induced models are recommended for inclusion in future studies. Biological assessment using serum samples is also necessary to provide comprehensive, evidence-based preclinical studies prior to conducting clinical research.

## Conclusion

Neuroinflammation is the leading cause of many NDS that result in brain tissue damage and memory impairment. In the brain, inflammatory responses can result in deleterious conditions such as Aβ and tau toxic aggregation. Perhaps, the most challenging concept in the field of research is to find an early diagnostic approach for AD as well as to determine an effective and reliable therapeutic intervention. A progressive increase in characteristic AD hallmark depositions was detected in the brain, spinal cord, and CSF of the AD-induced model from week 1 to week 3. This indicates that AD aggregations are not only confined to the brain but can be detected earlier in the biological fluid and spinal cord. In addition, our current study demonstrates the efficacy of a single ICV administration of PHM and CBX against the neuroinflammation-induced model of AD. Both have demonstrated neuroregenerative and neuroprotective actions through various signaling and anti-inflammatory actions. These findings imply that cell therapy and gap junction blockers against NDS may lead to new therapeutic approaches that can manage or alleviate AD symptoms affecting cognition, behavior, and function. On the other hand, further studies are needed to elucidate the harmful and beneficial effects of the therapeutic implementation of microglial-mediated therapy on NDS.

## Data availability statement

The raw data supporting the conclusions of this article will be made available by the authors, without undue reservation.

## Ethics statement

The animal study was reviewed and approved by the Koç University Institutional Animal Care and Use Committee (IACUC) in accordance with the directive 2010/63/EU of the European Parliament and Council on the protection of animals used for scientific purposes.

## Author contributions

MA, EÖ, and YG-Ö designed the experiment and reviewed and edited the final version of the current manuscript. MA, EÖ, NS, SS, CÖ, and JK performed the behavioral studies and conducted the whole experiment design. MA analyzed the results and wrote the manuscript. YG-Ö supervised the whole experiment. All authors who participated in the manuscript approved the final version.
